# Correlation of CK5 and EGFR with Clinicopathological Profile of Triple-Negative Breast Cancer

**DOI:** 10.1155/2014/141864

**Published:** 2014-10-23

**Authors:** Neelam Sood, Jitendra Singh Nigam

**Affiliations:** ^1^Department of Pathology, Deen Dayal Upadhyay Hospital, Hari Nagar, New Delhi 110066, India; ^2^Department of Pathology, Saraswathi Institute of Medical Sciences, Anwarpur, Hapur, Uttar Pradesh 245304, India

## Abstract

*Purpose.* Triple-negative breast cancer (TNBC) is defined by the loss of expression of ER, PR, and Her2*neu* expressions. The aim of this study was to examine the expression of the EGFR, CK5, and Ki-67 among triple-negative breast cancer cases and to correlate the expression of the basal markers with the clinicopathological prognostic parameters. *Materials and Methods.* Thirty-six female patients with TNBC based on ER, PR, and the HER2*neu* negativities were studied by immunohistochemistry for EGFR, CK5, and Ki-67 expression. Statistical analysis was done using the SPSS software version 20. *Results.* The mean and median ages were 45.18 years and 46.70 years, respectively. Infiltrating ductal carcinoma NOS was the predominant histopathological type (29/36 [80.6%]). The commonest histological grade was grade 2 (17/36 [47.2%]). Tumour necrosis was seen in 16/36 (44.4%) patients. Infiltrative margins were shown in 69.44% (25/36) cases. Ki-67 was positive in 80.56% (29/36) cases, 61.11% (22/36) were CK5-positive, and 86.11% (31/36) were EGFR-positive. The only significant positive association observed was between the CK5 and histological grade (*P* < 0.05). *Conclusion.* CK5 shows a statistically significantly correlation with TNBC histological grade. The majority of the specimens show EGFR expression. Therefore TNBCs could potentially benefit from EGFR-targeted therapeutic strategies.

## 1. Introduction

Triple-negative breast cancer (TNBC) is defined by the loss of expression of estrogen receptor (ER), progesterone receptor (PR), and human epidermal growth factor 2 (Her2*neu*) expressions and associated with biological aggressiveness and poor prognosis and two subtypes, basal and nonbasal, have been described [1]. TNBC and basal type are not synonymous but basal type has similar behavior as TNBC [2]. Basal type was defined as CK5-/6-positive and/or EGFR-positive, and nonbasal type was defined as having no expression of these two markers. Ki-67 is another marker being studied for its implication if any [1]. Bertucci et al. assessed the degrees of correlation and of homogeneity of the TN phenotype (IHC-based definition) and the basal subtype (gene expression-based definition) and observed that tumour classified as TNBC is defined by gene expression profile (GEP) as 71.51% basal (123/172 cases) and 28.49% nonbasal (49/172 cases). Conversely tumors were defined as basal by their gene expression profile and included 76.88% (123/160) TN and 23.12% (37/160) non-TN [3]. Basal type TNBC has high histological grade, p53 mutation, usually expresses basal cytokeratin (CK5/6, CK14, and CK17), epidermal growth factor receptor (EGFR) overexpression, significantly associated with Ki-67 labelling index, p53 expression, and BRCA1 expression, and showed a shorter overall survival than nonbasal type; however, GEP is considered to be the gold standard for basal type TNBC identification [4]. Rao et al. observed that 74% of TNBC showed the expression of the EGFR and/or CK5/6 [5]. CK5/6 and/or EGFR expressing breast tumors showed poorer prognosis in the longer term, and screening for those basal markers is important in determining prognosis and therapeutic strategies in TNBC. Therefore the basal cytokeratins and EGFR in immunohistochemical panel can be used to identify basal-like TNBC [4, 6]. TNBC rates are higher in women of African or Hispanic ancestry, premenopausal and younger age, women with early menarche, higher parity, younger age at full term pregnancy, shorter duration of breast feeding, higher body mass index, and higher waist to hip ratio especially among premenopausal patients [7].

## 2. Materials and Methods

The present study was done in DDU Hospital, Hari Nagar, New Delhi, during 2010 to 2013 and includes 36 female patients with TNBC based on the ER, PR, and the Her2*neu* negativities. Detailed clinical histories were taken and clinical examinations were done after taking their informed consents. The specimens were processed and fixed in 10% formalin and were examined grossly. Paraffin embedded sections were stained with the haematoxylin and eosin stain. The tumours were classified and graded according to the Nottingham modification of the Scarff-Bloom-Richardson system. The pathological variables were evaluated and pTNM staging was done.

The tissue sections were used for all the immunohistochemical analyses (ER, PR, Her2*neu*, EGFR, CK5, and Ki-67). The antibody clones which were used were for the estrogen receptor (BioGenex: Species mouse, clone 1D5, Isotype IgG1/kappa), the progesterone receptor (BioGenex: Species rabbit, clone SP2, Isotype rabbit IgG1), Her2*neu *(Diagnostic BioSystems: Species rabbit, clone BV5, Isotype IgG), EGFR (Diagnostic BioSystems: Species rabbit, clone SP9, Isotype IgG1/kappa), CK5 (BioGenex: Species rabbit, clone EPR1600Y, Isotype IgG), and Ki-67 (BioGenex: Species mouse, clone Ki-88, Isotype IgG1/kappa). All antibodies are ready to use. After bringing the section to water, antigen retrieval was done by boiling the section in either citrate buffer (pH 6, for CK5/6, Her*2neu*, and Ki-67) or EDTA buffer (pH 9, for ER, PR). For EGFR, antigen retrieval was done by heat induced epitope retrieval. Tissue controls were used for IHC assays. The section was washed with deionized water for 2 to 3 minutes followed by peroxide blocking for 30 minutes using 4% hydrogen peroxide in methanol; then the section was washed by Tris buffer (pH 7.5) for 2 to 3 minutes. Background blocking was done by horseradish peroxidase (HRP) for 10 minutes followed by primary antibody overnight. Next day wash with Tris buffer for 2 to 3 minutes was followed by secondary antibody (Biotin) for 30 minutes; then again wash with Tris buffer and tertiary antibody (Streptavidin) was applied for 30 minutes. Wash with Tris buffer twice and D.A.B. was applied for 10 to 15 minutes and reaction was stopped by using distilled water. Counterstain Hematoxylin was applied for 2 minutes and washed with distilled water.

The ER and PR scores were based on the proportions and the intensities of the stained nuclei, Her2*neu *score was based on the intensities and the proportions of the cells which showed membrane staining, cytokeratin 5 was scored as positive if cells showed any weak or strong cytoplasmic and/or membranous staining, EGFR was scored as positive if more than 1% of the tumour cells showed membrane reactivities, and Ki-67 positivity was quantified as the 10% percent of the positive nuclear staining of the tumour cells [5]. The data were entered and analyzed by using SPSS. The chi-square test was used to compare the association of the expressions of EGFR and CK5 and clinic-pathological characteristics of the tumours. The results were considered as statistically significant if the *P* value was <0.05.

## 3. Results

**Figure 1:**
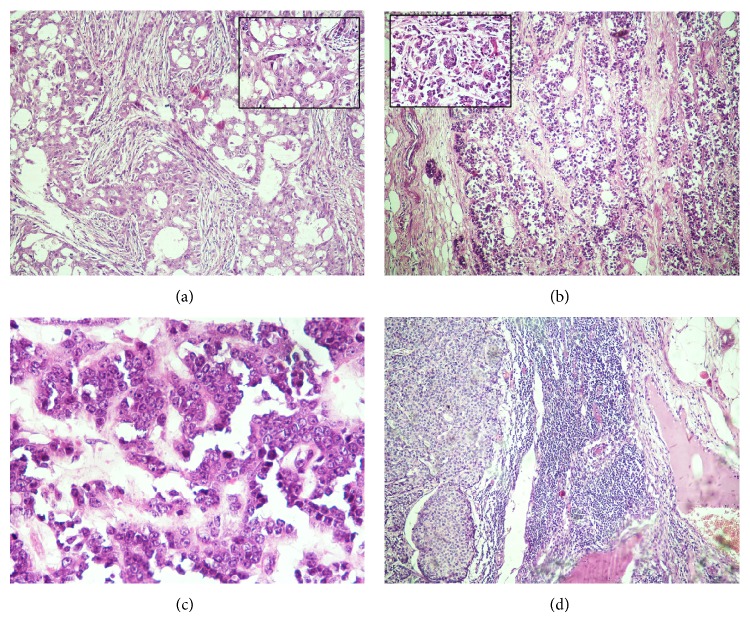
(a) Histological grade 1: tumour with tubular differentiation, regular outlines, and uniform nuclear chromatin (H&E ×100, Inset-H&E ×400). (b) Histologic grade II: tumours with cords, islands with tubular differentiation, open vesicular nuclei, and visible nucleoli (H&E ×100, Inset-H&E ×400). (c) Histologic grade III: infiltrative islands, with minimal tubular differentiation, marked variability in size and shape, open vesicular nuclei, and visible nucleoli (H&E ×400). (d) Invasive breast carcinoma involving lymph node (H&E ×400).

**Figure 2:**
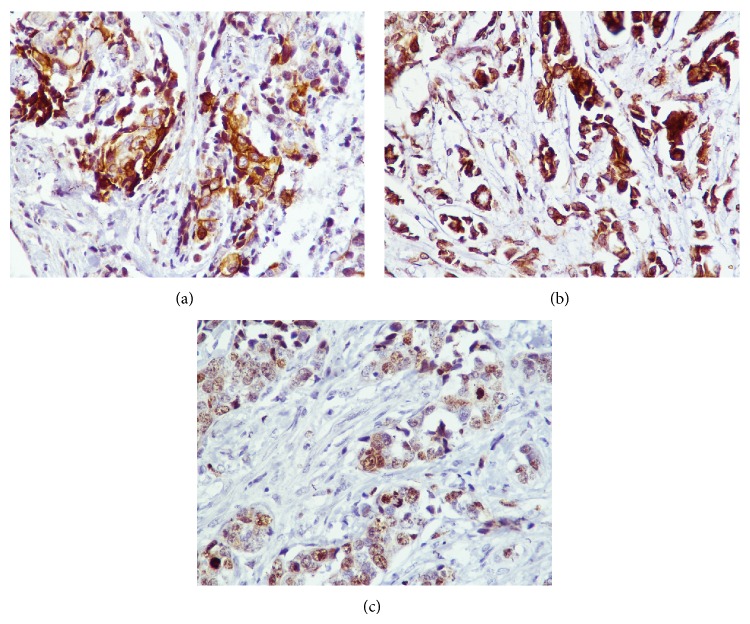
(a) Cytokeratin 5 positivity in tumour cells. (b) EGFR positivity in tumour cells. (c) Ki-67 positivity in tumour cells.

A total of 36 cases of infiltrating TNBC were included. The age of patients was ranged from 19 to 78 years with mean age of 45.18 years and median age of 46.7 years. Infiltrating ductal carcinoma NOS was the predominant histopathological type (80.56%, 29/36). In a majority of the patients, the tumour size was between 2.1 and 5 cm (50%, 18/36) followed by <2 cm (36.11%, 13/36). The commonest histological grade was grade 2 (47.22%, 17/36) followed by grade 3 (38.89%, 14/36) and grade 1 (13.89%, 5/36) (Figure 1). In grade 1 tumours, 2 cases show adenoid cystic pattern and 3 cases showed ductal carcinoma in situ (DCIS). In grade 2, 14 cases were associated with infiltrative borders, 3 cases were associated with DCIS, and 1 case was associated with intraductal papillomatous. In grade 3, 2 cases were associated with atypical medullary features, 1 case was associated with metaplastic carcinoma with osteoclastic giant cells, 1 case was associated with lobular carcinoma, and 2 cases were associated with DICS. Four cases of grade 3 and two cases of grade 2 tumours showed recurrence or metastasis within 5 years. Three cases of grade 3 and 2 cases of grade 2 died within 5 five years of surgery. Three cases of grade 1, 3 cases of grade 2, and 1 case of grade 3 were survived without any recurrences or metastasis. The rest of the cases were lost for follow-up. The commonest histological pTMN stage was stage II (44.44%, 16/36) followed by stage III (36.11%, 13/36) and stage I (13.89%, 13/36). The tumour necrosis was seen in 44.44% patients. Lymphocytic infiltrates were observed in 63.89% patients. 69.44% (25/36) cases showed infiltrative margins and 30.56% (11/36) cases showed pushing tumour margins. Ki-67 was positive in 80.56% (29/36) (Table 1). Out of the 36 TNBC cases, 61.11% (22/36) were CK5-positive and 86.11% (31/36) were EGFR-positive (Figure 2). We studied the association between the CK5 and EGFR expression with the clinicopathological prognostic parameters. A significant positive association was observed between the histological grade and CK5 but not with EGFR (Table 2).

## 4. Discussion

Breast cancer is a biologically heterogeneous disease and clinical outcomes of patients with the same diagnostic and clinical prognostic profiles are markedly different possibly due to molecularly distinct diseases into group into classes based mainly on morphology [8]. Breast cancers can be divided into five molecular subtypes which are two ER positive types (luminal A and luminal B) and three ER negative types (HER-2 expressing, basal-like, and normal breast-like) and have distinct clinical features, with markedly differing prognosis and clinical outcomes [8]. Nielsen et al. studied the immunohistochemical profile for breast basal-like tumors for the protein patterns that are characteristic of this subtype and examined the significance of these protein patterns using tissue microarrays and evaluated the prognostic significance of these findings [9]. They concluded that a panel of four antibodies (ER, HER1, HER2, and cytokeratin 5/6) can accurately identify basal-like tumors using standard available clinical tools and shows high specificity [9]. In the present study, the triple-negative breast cancer was correlated with age, tumour size, histolopathological type, tumour necrosis, tumour margin, lymphocytic infiltrate, lymph node status, tumour grades, tumour stages, and Ki-67. The commonest age group in the present study was ≤40 years (14/36) followed by >50 years (12/36); however, Tan et al. observed TNBC common in age >40 years and Rao et al. observed that mean age of TNBC is 46.8 years [5, 10]. In the present study CK5 and EGFR do not show significant correlation with age as observed by other studies [1, 5]. The commonest tumour size was ranged from 2 to 5 cm (18/36) and did not significantly correlate with basal markers as observed by other workers [1, 5, 10]. In the present study, the majority of the TNBCs were grade 2 (17/36) instead of grade 3 (10/36) but significant correlation with CK5 was noted unlike observation by Rao et al. where grade 3 was the commonest grade and was statistically not significantly correlated [5]. This could be possibly due to higher associated DCIS pattern and infiltrative margins in such cases. Low-grade TNBCs have also been observed by other too as observed in the present study [4]. The important histologic basal-like carcinomas are usually high histologic grade, pushing, noninfiltrative borders, and large zones of geographic or comedo-type necrosis; stromal lymphocytic infiltrates; scant stromal content; lack of tubule formations; marked cellular pleomorphism; high nuclear-cytoplasmic ratios; vesicular chromatin; prominent nucleoli; high mitotic indices; and frequent apoptotic cells, presence of metaplastic elements, and glomeruloid microvascular proliferation as also seen in the present study [4]. Infiltrative margins were more commonly observed than pushing margins in the present study; however, margins were statistically not significant which are also corroborated with results of other studies [5, 11]. Lymphocytic infiltrates and necrosis were present in 23 and 16 cases, respectively, and were statistically not significantly correlated with CK5 and EGFR. Other workers also observed lymphoid infiltrate which is statistically not significantly correlated with basal markers as in the present study; however necrosis shows statistically significant correlation [1, 5]. In the present study lymph node involvements were observed in 24 cases and were statistically not significantly correlated with CK5 and EGFR. Lymph node involvement is also observed by other studies [1, 5, 11] and is statistically not significantly correlated with basal markers [5]. In the present study, the commonest histological pTMN stage was stage II followed by stage III and stage I and histological stages were not significantly correlated with CK5 and EGFR. Kutomi et al. also observed that stage II is the commonest pTNM stage followed by stage I and stage III. Ki-67 was positive in 29 cases and was statistically not significantly correlated with basal markers similar to Rao et al. findings [5]. Foulkes et al. observed that TNBC which also expresses CK5/6, EGFR, or both may have a worse outcome than the TNBCs that are negative for both of these markers and the overexpression of EGFR is more common in the TNBCs than in other subtypes of breast cancer, and cetuximab, which is targeted against EGFR, is being further studied in combination with carboplatin [12]. To conclude, among the clinic-pathological factors in the present study, age, tumour size, histological type, tumour stage, tumour margin, presence of lymphoid infiltrate and necrosis, lymph node metastasis, and Ki-67 positivity did not show statistically significantly correlation with CK5 and EGFR; however tumour grade shows statistically significantly correlation with CK5 only but not with EGFR. TNBCs are heterogeneous group of breast tumours that possess distinctive pathological and clinical features and TNBC and basal-like breast carcinoma should not be used as synonymous. By using CK5 and EGFR, the basal-like breast carcinoma can be identified and the majority of the TNBC patients show EGFR expression; however CK5 shows the statistically significantly correlation with histological grade. Therefore basal-like breast cancers could potentially benefit from EGFR-targeted therapeutic strategies.

## Figures and Tables

**Table 1:** Clinicopathological variables.

Clinicopathological variables	
Age (years)	
Mean	45.18

Tumor size (cm)	
≤2 cm	13
2–5 cm	18
>5 cm	5

Histopathological diagnosis	
IDC, NOS	29
Mucinous carcinoma	2
Metaplastic carcinoma	1
Medullary carcinoma	3
Mixed ductal-lobular carcinoma	1

Tumor necrosis	
Present	16
Absent	20

Margin	
Infiltrative	25
Pushing margin	11

Lymphocytic infiltrate	
Present	23
Absent	13

Tumor grade	
Grade I	5
Grade II	17
Grade III	14

Lymph node metasatases	
Absent	12
1 to 3	14
>4	10

Tumour stage	
Stage I	5
Stage II	16
Stage III	13
Stage IV	2

Ki-67	
Positive	29
Negative	7

Immunohistochemical panel	
CK5+ EGFR+	22
CK5+ EGFR−	5
CK5− EGFR+	9
CK5− EGFR−	Nil

**(a):** 

Prognostic parameters	EGFR		*P* value	CK5		*P* value
	Positive	Negative		Positive	Negative	
Age						
≤40	13	1	>0.05	7	7	>0.05
41–50	8	2		6	4	
>50	10	2		9	3	

Tumor size (cm)						
≤2	11	2	>0.05	9	4	>0.05
2.1–5	15	3		11	7	
>5	5	0		2	3	

Histopathological diagnosis						
IDC, NOS	24	5	>0.05	17	12	>0.05
Mucinous carcinoma	2	0		1	1	
Metaplastic carcinoma	1	0		1	0	
Medullary carcinoma	3	0		3	0	
Mixed ductal-lobular carcinoma	1	0		0	1	

Tumour grades						
Grade I	4	1	>0.05	2	3	**0.052**
Grade II	17	0		8	9	
Grade III	10	4		12	2	

**(b):** 

Prognostic parameters	EGFR		*P* value	CK5/6		*P* value
Tumour stage						
Stage I	5	0	>0.05	2	3	>0.05
Stage II	12	4		11	5	
Stage III	12	1		7	6	
Stage IV	2	0		2	0	

Margins						
Infiltrative	22	3	>0.05	13	12	>0.05
Pushing margin	9	2		9	2	

Necrosis						
Present	12	4	>0.05	11	5	>0.05
Absent	19	1		11	9	

Infiltrate						
Present	20	3	>0.05	15	8	>0.05
Absent	11	2		7	6	

Lymph node metasatases						
Absent	11	1	>0.05	6	6	>0.05
1 to 3	11	3		11	3	
>4	9	1		5	5	

Ki-67						
Positive	25	4	>0.05	16	13	>0.05
Negative	6	1		6	1	
